# Orthopedic Surgery

**Published:** 1899-03

**Authors:** 


**Orthopedic Surgery.** By JAMES E. MOORE, M.D. Pp. 354. London: The Rebman Publishing Co. (Ltd.). Philadelphia: W. B. Saunders. 1898.

When another volume upon a small branch of general surgery is added to those which already exist we have a right to claim that it shall be justified by some evidence of originality in the method of treatment, or at least of such care and judgment in examining controversial points, and in fully and accurately setting forth the more recent additions to our knowledge, as shall make it something more than a numerical addition to an overburdened literature. Unfortunately, in both these respects, the work under review, in our opinion, signally fails. For example, in the section on knock-knee nothing is said about the possible *rôle* of the tibia and fibula in the pathology of that affection: pes cavus receives the consideration of only a few lines; while of the important and relatively little-known conditions, termed metatarsalgia and coxa vara the accounts are so meagre and inadequate as to be almost, if not quite, valueless. Nor do we find any compensation in the treatment of the more ordinary portions of the subject, which never rises above mediocrity and is often weak and unsatisfactory.

The publishers' work has been excellently done, and there is no lack of illustrations, well executed, which are liberally scattered through the letterpress.

